# Effects of increased space allowance on animal welfare, meat and ham quality of heavy pigs slaughtered at 160Kg

**DOI:** 10.1371/journal.pone.0212417

**Published:** 2019-02-15

**Authors:** Eleonora Nannoni, Giovanna Martelli, Giulia Rubini, Luca Sardi

**Affiliations:** Department of Veterinary Medical Sciences, University of Bologna, Ozzano Emilia, Italy; University of Illinois, UNITED STATES

## Abstract

Sixty barrows (Body Weight–BW- range: 23.9–160 kg) were allotted to two experimental groups (6 pens of 5 pigs each): the control group was kept at a space allowance of 1m^2^/head; the second group was kept at 1.3m^2^/head. Behaviour, growth parameters, carcass and meat quality were assessed, as well as fat and cured ham quality. Results showed that pigs raised at 1.3m^2^/head spent more time laying (particularly in lateral recumbency, P<0.01 and P<0.001, respectively) compared to pigs kept at lower space allowance. They also reduced the aimless exploration of the slatted pen floor (P<0.001) and increased overall expression of other, mainly active, behaviors (*e*.*g*., drinking, walking and standing, P<0.01). Pigs raised at 1.3m^2/^head showed higher final BW (P = 0.02), more favourable Average Daily Gain (ADG) and gain-to-Feed ratio (G:F) both during the last period of the trial (P<0.05 for both parameters) and over the entire trial (P = 0.01 for both parameters). No significant difference was observed between groups for carcass traits and the main meat quality attributes. Subcutaneous fat from green hams had higher α-linolenic acid content (P<0.01) in the group reared at greater space allowance. Green hams from this group lost less weight at trimming (P<0.01) and the resulting cured hams received better sensory evaluations (P<0.05). No difference was observed in fatty acid composition and unsaturation levels of the subcutaneous fat from cured hams. Our data suggest that heavy pigs intended for Parma ham would benefit from the adoption of higher individual floor space allowances, both in terms of animal welfare (increased possibility to rest) and of productive parameters, without having any detrimental effect on the suitability of the thighs for dry-curing or on the quality of the final product.

## Introduction

Floor space allowance can affect welfare and productive parameters of pigs [1–4]. European legislation [5] sets to 1m^2^/head the minimum floor space allowance for pigs over 110kg BW, but gives no further requirement for heavier animals, such as for example Italian heavy pigs (which reach and exceed 160 kg BW at the end of their production cycle [6]). Minimum space allowances (A) set by law [5] are calculated after the equation A = 0.030×BW^0.67^ where the coefficient of the equation (k) is set to 0.030 [3]. However, according to EFSA recommendations [4], higher coefficients should be used (k = 0.036 for pigs up to 110kg and k = 0.047 above 110 kg) in order to allow all pigs to lay down separately and at the same time [7]. Noteworthy, these minimum space requirements, which are based on lying behavior, do not consider the space needed for other fundamental behaviors (feeding, drinking, excretion, exploration, etc.) [8]. To the best of the authors’ knowledge, only Pastorelli et al. [9] have carried out a study aimed at calculating the space requirements for heavy pigs, despite the fact that Italian consumers perceive space allowance as the first structural factor affecting animal welfare [10]. As concerns studies on the impact of space allowance on carcass and meat quality, Serrano et al. [11] found a decrease in backfat MUFA (Mono Unsaturated Fatty Acids) as space allowance increased. Rossi et al. [12] found no differences in meat quality together with increased backfat thickness in heavy pigs raised at space allowances of 1.4 vs. 1m^2^/head. Conversely, the effects of different space allowances on cured ham quality have never been investigated. The aim of the present pilot study was to investigate the effects of two different space allowances (minimum space set by legislation vs. space calculated according to EFSA recommendations) on behavior, growth parameters, meat and ham quality of heavy pigs intended for Parma Ham production. It is hoped that this study could contribute to a better knowledge on allometric needs of heavy pigs and provide useful scientific background for a more inclusive legislation on pig protection.

## Materials and methods

The experiment was carried out in accordance with Directive 2008/120/EC on the protection of pigs. Animals were raised and sacrificed for commercial purposes. They were obtained from a commercial farm and raised in the facilities of the Department of Veterinary Medical Sciences of the University of Bologna (farm code: IT046BO065). Pigs were slaughtered at a commercial abattoir (CLAI, Ravenna, Italy). They were not subjected to any invasive procedure (blood sampling, etc.) therefore the trial did not fall within the field of application of the Directive 2010/63/EU on the protection of animals used for scientific purposes.

### Animals, housing and experimental design

Sixty crossbred Duroc × (Landrace × Large White) barrows were used. The average Body Weight (BW) at the beginning of the trial was 23.9 kg. Animals were homogeneously allotted to two experimental groups on the basis of their BW and litter of origin. Animals were raised until reaching the minimum age of 9 months and the BW of approximately 160 kg, according to the rules established for Parma Ham production [6].

Pigs were kept in small groups (5 animals per pen) on a totally slatted floor. Pens were located in temperature-and humidity-controlled rooms (22–24°C, 70–80% RH) equipped with a forced-air ventilation system. Each pen was equipped with a bite drinker and a collective stainless steel feeder. Environment was enriched by providing steel hanging chains. Commercial feed was offered twice a day as a meal, rationed at 9% of the metabolic BW. Every two weeks a sub-sample of animals (two pens) was weighted, and the feed allowance was adjusted to their BW, up to a maximum of 3.3 kg dry matter per pig, per day. Animals were allotted to two experimental groups, each comprising 6 pens of 5 pigs:

- the first group (control group) was kept at an individual space allowance of 1 m^2^/head (according to the minimum requirement set by European legislation [5];
- the second group (higher space allowance group)was kept at an individual space allowance of 1.3 m^2^/head (calculated according to Petherick and Baxter [7] and EFSA [4] recommendations on the basis of an average BW of 135 kg).

In agreement with the European legislation, the space occupied by the collective feeder was not considered as part of the floor space allowance per head.

### Behavioral observations

The behavior of all pigs (6 replications for each group) was videotaped (b/w cameras) over the diurnal hours (7:00 to 19:00) by means of a digital closed circuit system (Mesa, Arezzo, Italy). Cameras were mounted on a rail attached to the ceiling above the pens (approximately 3m above the ground). Pigs were videotaped over the diurnal hours once every two weeks, for a total of 11 videotaping sessions. Each session was automatically divided into 12 1-hour videos, which were stored in a dedicated hard drive, divided by day and camera. The stored videos were later examined by a single trained observer. The behavioral patterns were assessed by scan-sampling technique, which consists of sampling 10 seconds of video every 10-min of its duration and noting down the prevailing activity of each of the pigs in the 10 seconds of observation. Activities were recorded according to predetermined ethogram for heavy pigs, reporting the following behaviors: standing inactive, sitting inactive (dog-sitting), sternal recumbency, lateral recumbency, walking, eating, rooting/exploring the floor, social interactions. A detailed description of the behaviors observed in the ethogram is given in [13]. Data were then used to calculate the daily proportion of time spent performing each behavior by the pen of pigs. Results are expressed as the average per treatment of all the videotaping sessions across the trial.

### Growth parameters, carcass and meat quality

All pigs were individually weighed during the trial, at the approximate BW of 30, 60, 100 and 160 kg. Average daily gain (ADG) was subsequently calculated. Feed intake of every replication (i.e., pen) was recorded daily to calculate the feed conversion ratio (FCR). In order to comply with the required BW for Parma Ham production (on average 160 kg), pigs were slaughtered in two sessions. Data collection of growth parameters stopped on day 224, when 2/3 of the pens (4 entire pens per treatment) reached the average BW of 160 Kg and were slaughtered. The remaining pigs (2 entire pens per treatment) were kept under the experimental conditions up to the day in which they in turn attained the final body weight of about 160 kg. Pigs were transported to the abattoir, and slaughtered after a 15-h fast. Dressing out percentage was calculated, lean meat yield and subcutaneous fat thickness were measured by Fat-o-Meater (FOM-SFK, Copenhagen, Denmark). The pH value of the *Semimembranosus* (SM) and of the *Longissimus lumborum and toracis* (LL) muscles was assessed by means of a portable pH meter (model 250A; Orion Research, Boston, MA) at 45 min. post mortem. At 24 h post mortem, a second read oh the pH in the SM muscle was taken with the same instrument described above. Carcasses were dissected and the weight of the main commercial cuts (thigh, loin and shoulder) was recorded, to calculate their yield.

Color of the lean portion of the thighs (SM muscle) was measured using a Minolta Chromameter CR-400 (Konica Minolta optics INC., Japan) set with the D65 illuminant, according to the CIE Lab (L*, a*, b*) color space [14]. A sample was taken from the LL muscle of each pig and used to assess meat quality. Drip and cooking loss analysis were carried out on LD samples, according to the method proposed by Honikel [15]. Warner Bratzler Shear Force (WBSF) was measured on six cores from each cooked sample using an Instron Universal Testing Machine, model 1011 (Instron Ltd., England) fitted with a Warner-Bratzler (WB) device at a cross-head speed of 200 mm/min.

### Fat and ham quality

For each experimental group, 14 green hams were randomly selected and subcutaneous fat was sampled in the area overhanging *Biceps Femoris* (BF) muscle. Total lipids were isolated according to Folch [16] and, after methylation, fatty acid composition was determined by gas chromatography (HRGC8560 Series Mega 2 gas chromatograph; Fisions Instruments, Milan, Italy). Fatty acids were esterified using 5% methanolic hydrogen chloride. The fatty acid methyl esters were separated by gas chromatography using a Supelco SP- 2330 capillary column (length: 30m; internal diameter:0.25mm; film thickness: 0.2 μm; Supelco, Bellefonte, PA, USA). Injector and detector temperatures were kept at 220°C and 280°C, respectively. The column was programmed as follows: 140°C for 1 min; the temperature was then raised to 220°C (3°C/min) and held constant for 15 min. Fatty acids were identified by comparing the retention times of the peaks with those of known standards. Results are expressed as percentages of total fatty acids. The iodine number was determined according to the AOAC method [17].

All hams were dry-cured according to Parma Ham production rules [6] for 18 months, and followed during the entire dry-curing process. Green hams were weighted before and after trimming, after salting and at the end of the dry-curing period. Weight losses were calculated for each productive step.

At the end of the dry-curing process, the same 28 hams (14/group) analyzed for raw fat quality were deboned and a sample-slice (including BF and SM muscles), was taken transversally from the caudal portion of ham to the middle of the femoral bone impression. The slice was evaluated by a panel of trained experts. Evaluation was expressed according to [18] on a scale ranging from 1 to 10 (1 = absence of the trait; 10 = maximum presence) for the following parameters: texture, color dishomogenity and marbling for the lean portion; texture and thickness for the fat portion. An overall score was attributed as a global evaluation of the ham, expressed on a scale ranging from 1 to 10 (1 = very bad quality; 10 = optimal characteristics). With the same techniques described before (Minolta colorimeter), color of the SM muscle and of the subcutaneous fat was measured.

Subcutaneous fat samples (outer and inner layers) were taken from the skin-covered cured fat in the overhanging area of the BF muscle and analyzed by gas chromatography as described above (HRGC8560 Series Mega 2 gas chromatograph; Fisions Instruments) for fat from the raw thighs. Subcutaneous fat was analyzed for peroxide value [19]. Purified lipid samples were diluted in iso-octane and conjugated dienes and trienes were determined by measuring specific extinction at 232 and 268 nm (K232 and K268, respectively) [20].

As concerns the lean fraction, samples were taken from the BF muscle. Moisture and crude protein were analyzed according to AOAC methods [17], sodium chloride content and proteolysis index (non-protein nitrogen/protein nitrogen) were determined according to [21–22].

### Statistical analysis

Data were analysed using the STATISTICA 10 package [23]. Data were submitted to a linear model using individual space allowance as the main effect. The statistical unit was the pen for the growing (live weight, ADG, G:F) and behavioral parameters, the individual (pig or ham) for carcass, and ham quality data. For carcass and meat quality parameters, a mixed model with the pen as a random factor was tested. Since there were no differences with the results of the linear model, the results of the linear model are included in the manuscript. For nonparametric data (behavioral traits and sensory evaluation of hams), the Mann–Whitney test was used. The significance level for all statistical tests was set at P<0.05. All data are presented as raw means ± SE.

## Results

Table 1 shows the ethogram of the two experimental groups. General behavior was affected by space allowance, with pigs raised at 1.3m^2^/head spending an increased amount of time lying (and, in particular, in lateral recumbency, P = 0.007 and P = 0.001, respectively) if compared to pigs kept at lower space allowance. Besides, the group raised at increased space allowance reduced the time spent in aimless exploration of the slatted pen floor (P = 0.001) and increased the overall expression of other, mainly active, behaviors (such as drinking, walking and standing, P = 0.01).

**Table 1:** Behavior of heavy pigs raised at different floor space allowances. Data are expressed as a percentage of total observed behaviors.

	Floor space allowance			
	1 m^2^/head	1.3 m^2^/head	SE^1^	P-value
Replications, n.	6	6		
Sitting inactive	2.8	3.4	0.2	0.56
Lateral recumbency	29.4	35.4	0.9	<0.01
Sternal recumbency	33.7	32.3	0.8	0.20
Total recumbency	63.1	67.7	0.9	<0.01
Eating	8.9	9.2	0.6	0.91
Exploring of pen floor	22.0	15.0	0.7	<0.01
Social Interactions	2.2	3.1	0.3	0.52
Other (drinking, walking and standing)	1.0	1.6	0.1	0.01

^1^ Standard Error

Results on growth performance and carcass traits are reported in Table 2. Overall, pigs raised at increased space allowances showed more favourable growth parameters: higher BW at the end of the zootechnical trial (P = 0.02), more favourable ADG and G:F both during the last period of the trial (d 140–224, P = 0.002 and P = 0.03, respectively) and over the entire trial (P = 0.01 and P = 0.02, respectively). Tendential differences were observed also for BW at d 139 (P = 0.1) and for ADG in the intermediate period (d 82–139, P = 0.1). Differences between the experimental groups increased linearly, becoming more significant as the trial progressed: the differences in BW were 0.1 (n.s.),1.2 (n.s.), 3.5 (P = 0.1) and 8.2 (P = 0.02) kilograms at day 1, 81, 139 and 224 respectively. As a consequence, the difference in ADG and G:F followed a similar pattern: for ADG (P-value: n.s., 0.1, 0.002 in the three periods, P = 0.01 over the entire trial) and for G:F (P-value: n.s., 0.1, 0.03, in the three periods, P = 0.02 over the entire trial). No significant difference was observed between the groups in carcass traits (carcass weight, dressing out percentage, lean meat percentage) or in the yield of the main lean cuts (thigh, shoulder and loin). Backfat thickness was tendentially higher in the group raised at higher space allowance (P = 0.08).

**Table 2:** Growth performance and carcass qualityof heavy pigs raised at different floor space allowances.

	Floor space allowance			
	1 m^2^/head	1.3 m^2^/head	SE^1^	P-value
Pens, n° (replications)	6	6		
**Body Weight (BW), kg**				
Initial (d 1)	23.8	23.9	1.0	0.96
d 81	65.6	66.8	1.0	0.59
d 139	100.8	104.3	1.1	0.10
Final (d 224)	154.4	162.6	1.8	0.02
**Average Daily Gain (ADG), kg/d**				
d 1–81	0.516	0.529	0.006	0.27
d 82–139	0.606	0.647	0.012	0.10
d 140–224	0.631	0.686	0.010	<0.01
Overall ADG (d 1–224)	0.583	0.619	0.008	0.01
**G:F (Gain-to-Feed ratio)**				
d 1–81	0.345	0.353	0.004	0.28
d 82–139	0.269	0.287	0.005	0.10
d 140–224	0.206	0.223	0.004	0.03
Overall G:F (d 1–224)	0.256	0.271	0.004	0.02
Pigs, n°	30	30		
Carcass Weight (CW, kg)	134.8	136.7	1.0	0.37
Dressing out, %	83.0	83.5	0.2	0.13
Backfat thickness, mm	22.6	24.0	0.4	0.08
Lean meat, %	52.6	51.7	0.3	0.16
Thigh, %CW	24.2	24.5	0.1	0.19
Shoulder, %CW	14.9	14.9	0.1	0.87
Loin, %CW	10.6	10.9	0.1	0.15

^1^Standard Error

The main meat quality attributes (post-mortem acidification, water-holding capacity and tenderness) did not significantly differ between the experimental groups (Table 3). The only difference observed was in the color of SM muscle, with muscles from pigs raised at increased space allowance showing lower a* and Chroma values (P = 0.001 and 0.002, respectively), and tendentially higher hue values (P = 0.06).

**Table 3:** Meat qualityof heavy pigs raised at different floor space allowances.

	Floor space allowance			
	1 m^2^/head	1.3 m^2^/head	SE^1^	P-value
Pigs, n°	30	30		
pH 45min. LL	6.53	6.46	0.03	0.24
pH 45min. SM	6.53	6.50	0.003	0.70
pH 24h. SM	5.79	5.73	0.02	0.17
**Color SM muscle**				
L*	40.1	41.1	0.4	0.18
a*	9.5	7.9	0.3	<0.01
b*	3.0	2.8	0.1	0.51
Hue	0.31	0.35	0.01	0.06
Chroma	10.0	8.5	0.3	<0.01
Drip loss, %	1.48	1.59	0.04	0.19
Cooking loss, %	19.6	20.6	0.3	0.14
Shear force (WBSF), N/cm	5.1	5.4	0.2	0.34

^1^Standard Error

With respect to the quality of subcutaneous fat from green hams (shown in Table 4), no significant difference was observed in its fatty acid composition or in its overall unsaturation level. The only exception recorded concerns α-linolenic acid (C18:3), which was significantly higher (P = 0.01) in the experimental group reared at greater space allowance.

**Table 4:** Quality of subcutaneous fat of green hams from heavy pigs raised at different floor space allowances.

	Floor space allowance			
	1 m^2^/head	1.3 m^2^/head	SE^1^	P-value
Hams, n.	30	30	/	
Samples, n.	14	14	/	
**Fatty acid composition, %**				
C:14	1.60	1.59	0.02	0.87
C:16	23.6	23.7	0.1	0.87
C16:1	2.51	2.30	0.06	0.10
C18:0	12.1	12.2	0.2	0.73
C18:1	43.2	42.9	0.2	0.58
C18:2	12.9	13.2	0.2	0.42
C18:3	0.48	0.59	0.02	0.01
C20:4	0.57	0.59	0.02	0.33
SFA(Saturated Fatty Acids)	38.2	38.3	0.2	0.88
MUFA(Monounsaturated Fatty Acids)	47.5	47.0	0.2	0.29
PUFA(Polyunsaturated Fatty Acids)	14.3	14.7	0.2	0.33
**Iodine number**	64.6	65.2	0.3	0.38

^1^Standard Error;

Table 5 shows the weight losses of the hams during the entire dry-curing process, the instrumental color, and the results from the proximate and sensory analysis of the cured hams. Hams from pigs raised at higher space allowance lost less weight during trimming (P = 0.004) if compared to the control group. Instrumental color both of the lean and of the fat fraction highlighted no significant difference between groups. As concerns sensory evaluation, despite similar average results in the single parameters, the overall evaluation was significantly better (P = 0.012) for cured hams from the group kept at 1.3 m^2^/head.

**Table 5:** Weight losses, instrumental color, proximate and sensory analysis of dry-cured hams from heavy pigs raised at different floor space allowances.

	Floor space allowance			
	1 m^2^/head	1.3 m^2^/head	SE^1^	P-value
Hams, n.	30	30		
**Ham weight losses, %**				
After trimming	16.4	15.4	0.2	<0.01
After salting^♯^	3.78	3.91	0.07	0.33
Cured ham^♯^	32.4	31.9	0.5	0.59
**Samples, n.**	14	14		
**Proximate analysis**				
Moisture, %	59.8	59.6	0.2	0.67
Crude Protein, %DM	27.3	27.6	0.2	0.34
Proteolysis Index, %	25.5	25.8	0.5	0.79
Sodium Chloride, %	6.15	6.22	0.09	0.69
**Instrumental color**				
SM muscle				
L*	35.9	34.8	0.4	0.22
a*	12.9	13.0	0.2	0.79
b*	5.5	5.5	0.2	1.00
Hue	0.40	0.40	0.01	0.91
Chroma	14.1	14.2	0.2	0.85
Subcutaneous fat				
L*	73.2	74.6	0.3	0.06
a*	4.2	4.1	0.2	0.78
b*	4.0	4.2	0.2	0.61
Hue	0.77	0.79	0.03	0.77
Chroma	5.8	5.8	0.2	0.99
**Sensory analysis**				
Lean fraction				
Texture	7.9	8.1	0.2	0.67
Color inhomogeneity	1.7	1.5	0.3	0.77
Marbling	3.0	2.4	0.4	0.45
Fat fraction				
Texture	8.1	7.9	0.2	0.54
Thickness	6.9	7.3	0.3	0.78
Overall evaluation	6.6	7.1	0.1	0.02

^1^ Standard Error

^♯^ expressed as a percentage of trimmed weight

The quality of the fat from the dry-cured hams (acidic composition and oxidative status) is shown in Table 6. The two experimental groups had similar fatty acid composition and unsaturation levels. However, the extinction coefficient measured at 232nm was significantly higher in the control group than in the group kept at higher space allowance (P = 0.01).

**Table 6:** Quality of dry-cured hams from heavy pigs raised at different floor space allowances: Lean and fat portion.

	Floor space allowance			
	1 m^2^/head	1.3 m^2^/head	SE^1^	P-value
Samples, n.	14	14	/	
**Fatty acid composition (%)**				
C:14	1.37	1.36	0.02	0.73
C:16	22.8	23.0	0.2	0.75
C16:1	2.37	2.41	0.07	0.95
C18:0	11.5	11.5	0.2	0.94
C18:1	43.4	43.9	0.2	0.17
C18:2	11.3	11.0	0.2	0.40
C18:3	0.17	0.17	0.004	0.51
SFA(Saturated Fatty Acids)	37.0	37.1	0.3	0.94
MUFA(Monounsaturated Fatty Acids)	50.3	50.7	0.3	0.51
PUFA(Polyunsaturated Fatty Acids)	12.6	12.3	0.2	0.38
**Lipid oxidation**				
Peroxide value, meqO_2_	15	13	1	0.49
K_232_^♯^	5.0	4.0	0.2	0.01
K_268_^♯^	0.20	0.17	0.01	0.14

^1^Standard Error

^♯^ specific extinction coefficient measured at 232 and 268 nm according to [20]

## Discussion

Space allowance per head can be varied by varying either the pen size or the number of pigs in a pen. However, in the second case, an effect of group size on the experimental outcomes cannot be ruled out [24, 25]. Petherick [26] pointed out that group members time-share space, therefore the amount of free space to be shared is dependent not only on the space allowance per individual, but also on group size. Another confounding effect is that rearing systems offering increased space allowances often imply also an enriched environment (*e*.*g*., straw, access to an outdoor area) [27, 28]. Nevertheless, to the best of the Authors’ knowledge, in the literature the number of studies investigating the effects of space allowance without the confounding effect of group size and enriched environments is very limited. In the present study, we aimed at eliminating any bias due to group size (especially with respect to productive and behavioral data) by using different pen dimensions and keeping group size constant (5 pigs/pen).

Our results show that under our experimental conditions increased space allowance positively affected pigs’ behavior, with an increased degree of calmness (greater time spent resting in lateral recumbency, reduced time spent exploring the pen floor). The increased space allowance was calculated in order to give pigs the possibility to lay down all at the same time [7], and it may have prevented (or, at least, reduced) sleep disruption by other pen-mates. This, in turn, could have increased the pigs’ possibility to carry out longer (and/or synchronized) sleeping bouts. Vermeer et al. [8] used synchronized lying as an indicator of improved welfare, due to the fact that pigs, when possible, prefer to synchronize their behavior. In our study, also awake behavior was positively affected by increased space allowance: pigs showed a significant reduction in pen floor exploration, a behavior that, given the slatted floor and therefore the absence of rooting material, if over-expressed should be interpreted as a stereotypy [29,30]. Positive effects of increased space allowance on general behavior were observed also by Jensen et al. [24], who described increased exploration of rooting materials. Contrarily, Cornale et al. [31] observed no differences in behaviour, but lower fecal cortisol concentration in uncrowded pigs. However, it is worth noting that in both studies high space allowances were obtained by reducing group size. Lastly, as mentioned above, Vermeer et al. [8] observed an increased synchronization of resting periods and improved usage of functional areas, both behaviours indicating a reduced competition for resources (laying space or functional areas) and therefore an improved welfare of the animals.

As concerns growth parameters, overall ADG was in agreement with the recommendations for the Italian heavy pig production (i.e., approximately 600 g/d on the whole production cycle). Following specifications [6], this production requires animals of at least 9 months of age at slaughter, and weighing on average 160 ± 10 kg per lot. Rearing animals intended for dry-cured ham production therefore requires restricted feeding during the last phase, leading to sensibly less favorable growth parameters than those observed in most pig-producing countries, which typically market lighter (and younger) pigs. Regardless of the peculiarities in the Italian production system, our results show an improvement in growth parameters in the group raised at higher space allowances. Similar results were previously observed in pigs weighing up to 100kg [32, 33]. However, it’s interesting to note that in the present study differences between the experimental groups became more significant as the trial progressed. This aspect, although to the Authors’ knowledge has never been reported before, may indicate that space constraints may limit resources sharing by pigs (e.g., access to feed or to a resting area), and these effects might become more severe as animals grow up. A similar effect was observed also by Flohr et al. [34], who studied the effect of the removal of pigs from a group and observed that relieving stock pressure and providing additional floor space resulted in improvements in gain, suggesting that the allometric method is valid also for the prediction of floor space needs for heavy pigs. With respect to overall growth parameters, the only similar study carried out on Italian heavy pigs [12] found tendentially higher final BW in pigs kept at high space allowance (however, in the mentioned study group size was not constant). Other studies were carried out using lighter pigs (below 100 kg BW): Jensen et al. [35] found a tendential improvement in ADG for pigs kept at higher space allowances, whereas Gonyou et al. [36] separately analyzed the effects of group size and space allowance and found that both reduced group size and increased floor space area lead to higher ADG. It should however be noticed that, given the limited number of animals used in the present study and the small group size (due to the behavioural observations they were subjected to), the improvement in growth parameters should be regarded as indicative and might not be as evident if transposed to on-farm environment. Despite these limitations, under our experimental conditions the animals kept at lower space allowance took on average 7 days more (234.5 vs. 227.5 days) to reach the same slaughtering BW (162.5kg).

Carcass traits were not affected by space allowance, despite the differences we observed in growth parameters. Given the specialty nature of the derived meat products, in this kind of study the assessment of carcass, meat and subcutaneous fat quality is necessary (even when differences in such parameters are not to be expected), in order to show that the typical features of the raw material (and, therefore, of the dry-cured products) are not affected. This is particularly true when differences in growth rates (which could in turn affect carcass, meat and fat quality) are observed.

In this study, overall meat quality was not affected by the experimental treatment. In her review on stress reactions at slaughter (regardless of BW), Terlouw [37] hypotesized that pigs reared in an enriched environment (with larger pens and straw bedding), being less active and reactive during transport and lairage, might have higher glycogen levels at slaughter, possibly resulting in decreased pH. In the present study, the only difference observed was in SM muscle color, with muscles from pigs raised at increased space allowance showing lower redness and lower Chroma values (*i*.*e*., lower difference from a grey of the same lightness) if compared to the control group. However, these differences are of difficult interpretation considering that no alteration in the rate of postmortem glycolysis was found between the experimental groups.

Overall, fatty acid composition of the raw subcutaneous fat (total MUFA, PUFA and SFA content) did not differ between the experimental groups, despite the differences in growth parameters described above. The only significant difference was detected for linolenic acid content, which reached greater values in the group kept at higher individual space allowances. However, these differences did not affect the overall level of unsaturation (*i*.*e*., the oxidative stability) of green hams, as highlighted by iodine number, which did not differ between the experimental groups. Linoleic acid content and iodine number fell within the limits imposed by Parma Ham production rules [6]. The information available on the influence of housing density on fatty acid profiles is scarce [38,11] and differences in linolenic acid content were never observed before, although the mentioned papers studied different housing systems and lower slaughtering BW. In particular, Patton et al. [38] observed that adipose tissue from pigs provided less space was more saturated and was composed of higher percentages of PUFA. Such differences were not observed in the present study, similarly to Serrano et al. [11], who observed only differences due to gender and no difference due to housing density in the fatty acid composition of the outer layer of the subcutaneous fat.

Trimming is a traditional procedure aimed at obtaining the desired shape of the thighs. The lower relative weight loss after trimming observed for hams deriving from pigs kept at the higher space allowance can be attributable to the higher fat thickness of the thighs themselves depending, in turn, on the tendentially higher subcutaneous fat thickness observed in the pigs of this group, despite the similar body weight at slaughtering and carcass weight.

The higher overall sensory evaluation obtained by dry-cured hams from this group might be due to the numerically lower marbling score and higher fat thickness in these hams if compared with the control group. In fact, excessive marbling in the lean fraction may slow down salt penetration [39], whereas a minimum thickness of the subcutaneous fat is prescribed by the production specifications [6] in order to protect the lean portion from excessive proteolysis during the long dry-curing process.

Chemical analysis of the lean fraction (moisture, proteolysis index and salt) confirmed the compliance of the dry-cured hams to the production specifications, without differences between the experimental groups, with the only exception of the extinction coefficient measured at 232nm (K_232_), which was significantly lower in the group kept at higher space allowance. This absorbance is due to the formation of conjugated dienes (CD) deriving from the oxidation of linoleic acid. The formation of CD, which parallels the production of hydroperoxides, occurs at the early stages of lipid oxidation [40, 41]. These intermediates are then expected to decompose to secondary products, which, in the present study, have not been investigated. Although such a difference could be positively regarded (since it indicates a reduced presence of primary oxidation products), it is difficultly ascribable to the different space allowances at which animals were kept. Overall, it is worth noting that the slight difference in fatty acid composition (of the green hams) and in lipid oxidation (after dry-curing) did not impair the suitability of the thighs for dry-curing or the quality of the final product. Besides the positive effects on animal welfare due to the fact that pigs are allowed to rest more comfortably, it should be highlighted that the adoption of higher individual floor space allowances can improve the production parameters even in restricted-fed animals, without having any detrimental effect on meat or ham quality.

## Conclusions

The absence of specific requirements for individual space allowance of heavy pigs (about 160 kg BW) can result in the adoption of the minimum floor area required by the EU law (1 m^2^/head for all pigs weighing more than 110 kg), although such a provision does not keep into account the basic needs of heavier pigs especially in terms of space for resting. Our data suggest that, in the case of Italian heavy pigs, the adoption of higher individual floor space allowances (1.3 m^2^/head) can have positive effects on animal welfare (increased possibility to rest) and improve their productive parameters, without negative effects on the suitability of the thighs for dry-curing or on the quality of the final product. However, given the experimental conditions under which the present trial was carried out (small group size), further on farm investigations might be necessary to corroborate the differences observed in terms of growth parameters and behavioural traits.
